# University commitments to a sustainable food system: a content analysis of UK higher education institutions food sustainability policies

**DOI:** 10.1017/S1368980025101055

**Published:** 2025-09-11

**Authors:** Claire Blennerhassett, Bethan Turton, Emma Reeder, Stuart Baker

**Affiliations:** Edge Hill University, Faculty of Health, Social Care and Medicine, Ormskirk, United Kingdom

**Keywords:** Food sustainability, Policy, Public health, Food waste, Animal source foods

## Abstract

**Objective:**

The food system is a major contributor to the global burden of disease, ecosystem destruction and climate change, posing considerable threats to human and planetary health and economic stability. Evidence-based food policy is fundamental to food system transformation at global, national and local or institutional levels. This study aimed to critically review the content of universities’ food sustainability policy documents.

**Design:**

A systematic search of higher education institutions’ policies, using targeted websites and internet searches to identify food sustainability policy documents, was conducted between May and August 2023. A quantitative content analysis of the identified documents was conducted independently by multiple researchers using a coding template. Inconsistencies in coding were subsequently checked and amended through researcher consensus.

**Setting:**

163 UK higher education institutions.

**Participants:**

N/A.

**Results:**

Approximately 50 % of universities had a publicly available food sustainability policy. The most common food sustainability commitments therein were communication and engagement (95·2 %), food waste (94·0 %) and quality standards and certification (91·7 %). The scope of policy commitments varied between institutions; however, comprehensive documents included multifaceted commitments tackling more than one dimension of sustainability, for example, waste mitigation strategies that tackled food insecurity through food redistribution. Few (17·9 %) policies included a commitment towards research and innovation, suggesting university operations are considered in isolation from academic and educational activities.

**Conclusions:**

Multifaceted policy commitments are capable of uniting numerous food-related actions and institutional activities. As such, they are likely to support food system transformation, with broader positive outcomes for the university, students and the wider community.

The food system, comprising ‘all the elements (environment, people, inputs, processes, infrastructure) and activities that relate to the production, processing, distribution, preparation and consumption of food, and the outputs of these activities’^((1), p23)^, is complex, with diverse social, economic and environmental impacts. First, food production and consumption are major contributors to diet-related diseases, societal injustices and environmental damage, posing a threat to human, animal and planetary health. Dominant intensive farming practices have resulted in antibiotic resistance, soil degradation, environmental pollution and biodiversity loss, along with negative health outcomes for workers, nearby inhabitants and consumers^(2)^. Furthermore, high consumption of ultra-processed food, primarily dominating low socio-economic households in high-income countries^(3)^, is associated with increased risk of obesity, metabolic syndrome, CVD, depression and all-cause mortality^(4)^. From a societal perspective, the food system is also a major source of gender inequality^(5)^, power imbalances between food system actors^(6)^ and disregard for farmed animal welfare^(7)^ with wide-reaching consequences. As such, there is a need for policies that address power imbalances, empower vulnerable actors and protect human and non-human animals within the food system^(8)^.

Universities are in a strong position to drive the transition towards a just policy and governance landscape, as they play an instrumental role in producing the evidence underpinning a sustainable food system – one that ‘delivers food security and nutrition for all in such a way that the economic, social and environmental bases to generate food security and nutrition for future generations is not compromised’^((9): 1)^. This includes research to address critical issues in relation to (i) agriculture (e.g. yield gap) and food production, (ii) consumption patterns that drive production of high-impact foods and (iii) inequalities and imbalances within the food system, offering three main perspectives for achieving a sustainable food system^(10)^. First, efficiency-oriented solutions, driven primarily by technology innovations such as digital agriculture (e.g. artificial intelligence), new physical systems (e.g. advanced robotics) and gene technology (e.g. genome editing)^(11)^, which have been criticised for being a capitalist fix that neglects more ambitious change^(12)^. Second, strategies to reduce the demand for meat and dairy products (demand which is arguably generated by producers)^(10)^, with researchers searching for the elusive optimal way to change consumer behaviour. Finally, food system transformation, focusing on structural changes to address imbalances between actors within the food system^(10)^. The latter places responsibility on the system rather than individuals^(10)^ and attempts to harmonise production and consumption goals^(13)^, offering greater opportunity to consider possible unintended consequences and trade-offs between policy objectives.

Universities also have considerable responsibility for developing a workforce that considers the long-term future of the economy, ecology and equity among all communities, through commitments to education for sustainable development^(14)^. Despite this, a recent commentary from the scientific community has argued that universities are at risk of undermining the evidence base and perpetuating the status quo, as many university’s food provisions remain dominated by animal products^(15)^, which are disproportionally responsible for many environmental issues, including greenhouse gas (GHG) emissions^(16)^ land use change, water use and biodiversity loss^(17)^. Furthermore, initiatives implemented in catering facilities (e.g. meat-free Mondays) and departments (e.g. plant-based catering for events) are often disconnected from institutional planning, target-setting and monitoring^(17)^, potentially resulting in inconsistencies between departments or with institutional positionality, which could be a source of confusion among consumers.

Examples of how food sustainability (FS) policy could improve institutional food provisions appear to be scarce; however, a case study within the University of California shows promise for meeting climate mitigation targets by combining several FS policy interventions. In this study, the authors^(18)^ employed modelling techniques to estimate the change in GHG emissions of eight individual policy targets and two combined approaches. Reductions in emissions from food provisions varied from 0·7 % for swapping from beef to blended (70 % beef, 30 % plant) burgers to 21·2 % for introducing tray-less dining. The latter reductions were associated with reduced plate waste. More impressive was the multidimensional approach of tray-less dining, a ban on sugar-sweetened beverages, composting and the inclusion of food and the environment in academic content (assuming dietary change persistence through graduation), which could produce up to 41·9 % reduction in emissions. This is proposed to be more than double the reduction of GHG emissions needed to reach their 2025 target; however, changes in sales data are needed to quantify the actual impact of these policy targets on consumer behaviour and environmental outcomes.

In two recent policy review studies, notable weaknesses and discrepancies in the extent to which university policy is commensurate with climate emergency declarations^(19)^ and university activities^(12)^ were brought to light. First, the proportion of universities with a sustainability policy that included a food theme was low, equating to just 35·3 % of a representative sample of 17 UK universities^(12)^ and 30·9 % of all 165 UK higher education institutions^(18)^ at the time of the reviews. This is disappointing, given that UK institutions offered food to a student population of over 2 million people^(20)^ during a similar period, likely resulting in a not insignificant contribution to their Scope 3 emissions^(18)^. These emissions are all indirect emissions, not included in Scope 2 (i.e. indirect emissions from the generation of purchased energy), which occur in the value chain of the reporting company^(21)^. Furthermore, O’Neill and Sinden^(12)^ noted how research that questions the dominant modes of consumption is often missing from university sustainability strategies. This disconnect between universities as sites of radical research and as spaces for sustainability practices has been termed the ‘cognitive-practice gap’^(12)^. Finally, Hoolohan *et al.* ^(19)^ identified that only 54·9 % of those universities with an FS policy recognised the importance of meat reduction to mitigate GHG emissions, and just 9·8 % of these included a measurable target, casting doubt on the meaningfulness of the policies for addressing failings in the food system. It is important to note that neither study explored the wider dimensions of food provisions that threaten FS (e.g. food waste), pointing to an important gap in the literature. Therefore, this research aimed to conduct a comprehensive review of UK universities’ FS policies for two main purposes: (i) to capture the range of FS commitments covered within the policies and (ii) to share examples of good practice that could be used to influence future FS policy within the higher education sector.

## Methods

**Fig. 1:**
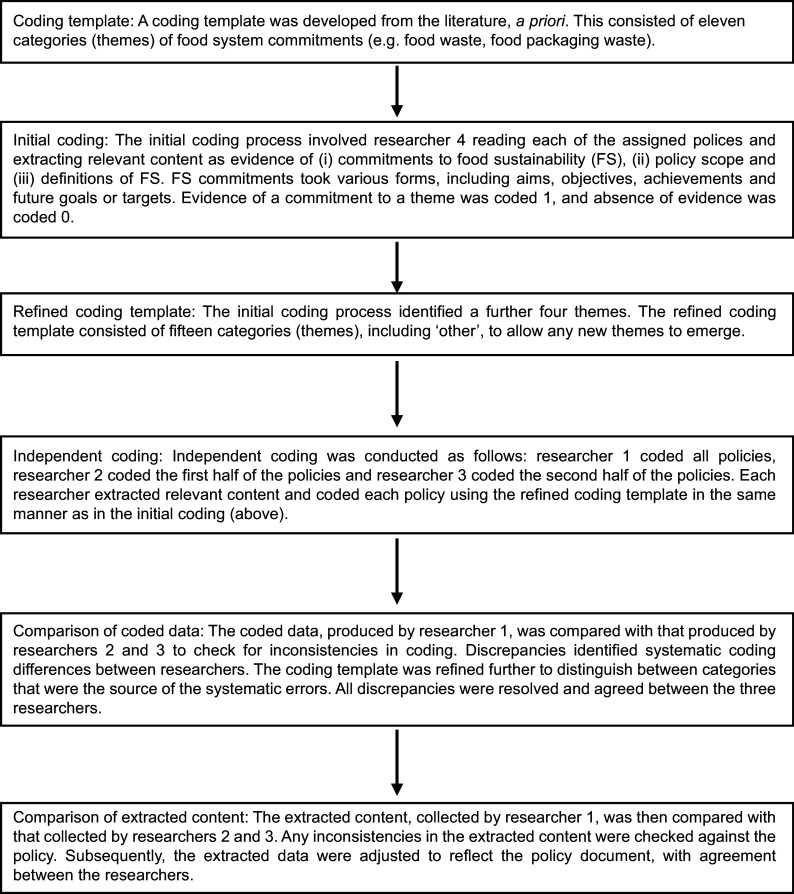
Steps involved in the coding process and during the reliability checks.

Content analysis, favoured for its flexibility in analysing textual data in a systematic and rigorous manner^(22)^, was employed to explore the content of universities’ (and other higher education institutions) publicly available FS policies. A quantitative approach to the content analysis was selected to provide a robust and reproducible assessment of the prevalence of specific FS themes across the dataset. This included establishing a coding system, coding the data, checking the reliability of the coding and adjusting the coding system as necessary (Fig. 1), before analysing the coded data and writing up the results^(22)^.

### Policy search

A list of UK universities was generated from the *Times Higher Education* rankings^(23)^. A search of the institutions’ websites was conducted to identify how many had a current and publicly available FS policy document. The search tool function, using the terms ‘food policy’, ‘food sustainability policy’, ‘catering policy’, ‘environmental food policy’ and ‘environmental catering policy’, was employed throughout the search. The search focused solely on publicly available FS policies as transparent policy dialogue is considered an integral component of a sustainable food system^(24)^. Where a policy was not identified, an internet search, using Google as the search engine, was conducted using the same terms in combination with the name of the institution. The search was completed between May and August 2023 to provide a snapshot of available policies within that period. All identified policies were downloaded and checked independently against the inclusion and exclusion criteria by the first (researcher 1) and last (researcher 4) author.

### Inclusion and exclusion

To provide a comprehensive analysis of universities’ commitments to FS, it was important to include all relevant FS policy documents. For this research, food policy was considered ‘a wide range of actions and decisions concerning the production and processing of food, its impact on public health and well-being, the environment, and natural resources’^((24): 215)^, involving both ‘consumers and producers’. Therefore, all documents identified during the search that specified an action or decision related to the production and processing of food, with implicit or explicit reference to consumers or producers, were included in this study. These included documents with and without policy in the title. The latter were labelled in a variety of ways, namely: food ‘strategy’ (*n* 1), ‘road map’ (*n* 1), ‘commitment’ (*n* 1), ‘plan’ (*n* 4), ‘objective’ (*n* 1), ‘action plan’ (*n* 1) and ‘charter’ (*n* 1). Excluded from the analysis were online FS materials that were focused on providing information (*n* 16), for example, ways to reduce food waste, rather than university actions or commitments towards FS. Policy documents older than 10 years (*n* 6) were also excluded, as they likely reflected historic documents that no longer reflected the institutions’ position on FS.

### Coding template

A template (Appendix A: see online supplementary material, Supplemental Material 1) for analysing the content of the policies was developed from the sustainable food system literature (*a priori*), with consideration of the three pillars of sustainable development, namely, social, environmental and economic^(25)^. This template consisted of eleven categories.

### Initial coding

To assess whether the template was able to capture the full range of FS commitments, researcher 4 coded a sample of FS policies, making a note of any commitments that did not appear to align with the original categories. Institutions with commitments to a theme were coded 1, and content from the policies was extracted as evidence. Those without a specific commitment were coded 0. During the coding process, additional themes were identified, resulting in a refined template of fifteen thematic categories (Appendix A).

### Independent coding and reliability checks

All policies were subsequently coded independently, using the refined template, by the first three authors (researchers 1–3). Following this, the researchers met to check the reliability of their coding by comparing their independently coded data and extracted content from the policies. Together, this identified between 1·2 % (meat, dairy products and eggs and fish) and 45 % (fruit, vegetables and plant-based ingredients) variance between coders. The latter, sizeable difference was due to systematic discrepancies in the coding process (e.g. one researcher coded content related to local and seasonal food to the ‘fruit, vegetable and plant-based ingredients’ theme, despite no reference to the food group, and another coded this content as procurement). This resulted in a further refinement of the template to distinguish between general procurement commitments to source food in a socially and environmentally way, from specific targets for purchasing local and seasonal produce (including both fruit, vegetables and other fresh produce, e.g. meat, fish, grains). In instances where the extracted content from individual coders did not match, the researchers referred to the original policy to identify if both extracts were present in the policy and relevant to the assigned category. Any inconsistencies were adjusted to reflect the policy, with agreement between the first three researchers.

### Data analysis

The coded data were analysed to quantify the prevalence of policy commitments for each theme across the dataset and the number (and percentage) of policy commitments per institution. The latter was used to calculate the average number of policy commitments using median and interquartile, as the data were not normally distributed (Shapiro–Wilk, 0·952, *P* = 0·003). The extracted narrative data were used to provide context as to the nature of the commitments for each theme, highlighting any variability in the quality of commitments and areas of good practice. In this instance, good practice was considered as commitments that extended beyond legislative compliance and accepted practices, based on common sense^(12)^, focusing on transformative or multidimensional approaches, towards a sustainable food system.

## Results

### University and policy characteristics

Of the 163 UK universities listed in the *Times Higher Education* rankings, 84 (51·5 %) had a recent and publicly available FS policy (Table 1). These institutions had a combined population of ∼1·3 million students, representing 65 % of the UK student population. Approximately 50 % of the policies available specified a review date between 2023 and 2028; however, one policy expired in 2022, and a further 27·4 % did not specify a review frequency or date.

**Table 1:** Food sustainability policy characteristics

	Number	%	Details
Food sustainability policy			
No	57	35·0	
Excluded > 10 years old	6	3·7	
Excluded information page	16	9·8	
Yes	84	51·5	
Publication date:			
Yes	78	92·9	Ranged from 2017 to 2023
No	6	7·1	Policy webpage (*n* 5), due review 2025 (*n* 1)
Review details:			
Expired	1	1·2	Policy period 2021 to 2022
Expiration in year of review	15	17·9	For review in the current year (2023)
Expiration after review	26	31·0	Review date range: 2024 (*n* 14) to 2028 (*n* 1)
Review frequency	19	22·6	Quarterly (*n* 1), bi-annual (*n* 1), annual (*n* 17)
Not reported	23	27·4	Some policies referred to the review process

The majority (96·4 %) of policies provided some indication of the scope of the publication. This included reference to the operational activities (i.e. procurement, processing) or products (e.g. food, beverages) it covered, university and non-university outlets the policy applied to and the consumers it related to, but few policies referred to all these domains (Table 2). Some institutions also specified outlets outside the scope of the policy (e.g. vending machines). Less than a fifth (*n* 14) of policies included a definition of FS or a sustainable food system. Of these, six acknowledged that there is no agreed-upon definition. As such, the definitions varied considerably between policies: from a brief definition sourced from the Department for Environment, Food and Rural Affairs (‘food which is healthier for people and the planet’) to the broad definition from the Sustainable Development Commission, which refers to social, economic and environmental considerations, including safe, healthy and nutritious food for consumers, viable farmer livelihoods, support for rural economies, employee safety, animal welfare and a broad range of environmental factors. Other policies did not include a source for their working FS definition.

**Table 2.** Scope of the food sustainability policies

Item	Examples
Products and services	All food and beverages Products and services, we provide Food served by food partners
Customers	Students, staff and visitors All aspects of catering to students, staff, visitors, conference delegates and leisure guests where practicable
University outlets	Owned shops and cafés All catering outlets and food served for events (internal and external) Vending services under the university’s managed catering contract Catered accommodation, retail outlets, bars, catering, events and mobile catering Event catering and bars, delivered hospitality, restaurants and cafés, residential catering Catering and hospitality, retail and Students’ Union Food and drink outlets, hospitality business and the nursery The university’s owned catering outlets
Other outlets	Outlets in departments operated by the private sector Third-party outlets Contracted catering companies
Operations	Procurement, preparation, provision, food waste, waste management and education Produced, processed and traded All stages of the food delivery chain (procurement to consumer. Food/materials, waste management and communication to consumers
Exclusions	Students’ Union Vending operated by an independent contractor Individual departments, societies, activities and events Brand/franchised outlets have their own sustainability goals. The supermarket and Student Association

### Policy commitments

The median (interquartile range) number of policy commitments was 11 (3), indicating that universities overall made a pledge towards almost three-quarters of the identified policy themes. The prevalence of policy commitments for each theme, along with supporting extracts from the publicly available FS policies, is presented in Table 3. This highlights that the most common policy commitments were for communication and engagement (95·2 %), food waste (94·0 %) and quality standards and certification (91·7 %). This was followed closely by commitments related to meat, eggs and dairy products, local and seasonal, procurement, non-food waste, fish and nutrition, health and well-being, which were present in more than three-quarters of policies. The least common commitments, present in less than half of the policies, were research and innovation (17·9 %), palm oil (29·8 %) and water resources (45·2 %).

**Table 3:** Food sustainability policy themes and supporting policy extracts

Theme	Number	%	Supporting policy extracts	
			Detailed	Vague
Communication and engagement	80	95·2	Communicate our aims and commitment to serving sustainable, regional food to our customers. Engage clients, stakeholders and students on a quarterly basis by showcasing our new products and actively seeking feedback before new menus are finalised. Raise employee awareness of relevant environmental and social effects of purchasing through appropriate training (Policy 134).	Promote the use of organic ingredients and products (Policy 198).
Food waste	79	94·0	Divert any surplus food fit for consumption through redistribution apps or Zero Food Waste or similar schemes (KPI: 750 unwanted meals redistributed per annum). Ensure all remaining food waste is disposed of through digestion, composting or energy recovery and no material will be sent to landfill. Ensure surplus coffee grounds are collected for processing into biofuel (Policy 108).	Managing our waste effectively so we can reduce, re-use and recycle where possible (Policy 116).
Meat, eggs and dairy products	75	89·3	Reduce the amount of beef we serve by 35 % annually, culminating in the complete removal of beef products in 2025–26. By 2022–2024, all beef and lamb will be from UK providers only. At least 80 % of the meat purchased will be higher welfare. This will increase by 5 % per year. Monitor and reduce the amount of meat (particularly processed meats), replacing this where necessary with pulses, beans and other sources of protein (Policy 106).	Serve high-welfare meat, dairy products and eggs (Policy 132).
Fish	73	86·9	Eliminate the use of fish from the Marine Conservation Society’s ‘Fish to Avoid’ list, and where possible, aim to only serve fish with a ‘best choices’ rating from the MCS Good Fish Guide, based on information at the time of purchase. Use diverse species of white fish to reduce pressure on sensitive stocks. Consider other options for increasing the sustainability of fish, including opting for pole and line caught fish, promoting consumption outside of the ‘big five’ (cod, salmon, tuna, haddock, prawns) and reducing or eliminating the consumption of carnivorous aquaculture species such as shrimp, prawns and salmon in favour of lower-impact aquaculture species such as tilapia (Policy 104).	Serving sustainably caught fish from the UK (Policy 112).
Quality standards and certification	77	91·7	All the tea, coffee, sugar and bananas provided to be Fairtrade (subject to supply). Continue to provide at least 40 % of our chocolate confectionery products as Fairtrade or ethically sourced. All fruit and vegetables to be Red Tractor assured. Where appropriate, the RSPCA monitored Freedom Food standard or equivalent will be used. We will monitor the amount of fruit and vegetables we purchase under schemes such as LEAF-Marque certification and/or Organic certification that aim to reduce their negative impacts on the environment (Policy 108).	Providing foods from certified accredited sources with high welfare, sustainability and ethical standards and avoiding GM ingredients (Policy 128).
Local and seasonal	75	89·3	Use of appropriate seasonal fruit and vegetables. Where possible, we will use local suppliers, with local defined as within a 50-mile radius. Producers from further away are permitted when no local equivalent can be found or dependent on the product required. Using local, seasonally available ingredients as standard to minimise energy used in production, transport and storage (Policy 140).	Include more seasonal and local produce (Policy 132).
Plant-based	60	71·4	We hold monthly ‘Eat Well Wednesdays’ where 70 % of the menu is suitable for vegetarians and reduce the meat in the other dishes that are served, replacing where necessary with pulses, beans and other sources of protein that are not of animal origin and our selections in other outlets are heavily meat free (Policy 168).	Increase the proportion of plant-based food offering (Policy 202).
Palm oil	25	29·8	Review the content of palm oil in all products bought, and create a matrix detailing all products. Review whether the palm oil is from sustainable sources and whether there is a non-palm oil alternative. Commit to a palm oil statement with the university, including reviewing the suitability of RSPO-certified products and connection with charities such as the Sumatran Orangutan Society (Policy 176).	No palm oil is purchased for use in our cooking (Policy 186).
Non-food waste	74	88·1	Implementation of a 20p discount for customers who bring their own reusable cup, thus reducing the number of disposable cups sold. The removal of all plastic bottles from our Hospitality Service, with water provided in recyclable glass bottles instead. All cling film has been removed from our Hospitality Service, with the implementation of washable and reusable trays and food coverings. No plastic cutlery on site, only wooden or cutlery made from plants is used. All plastic straws in every unit have been replaced with paper straws. Increased the number of water fountains on campus to support the reduction in single-use bottled water (Policy 176).	Reduce packaging, or use recyclable or compostable packaging where practicable (Policy 170).
Resources				
Energy/Water	49/38	58·3/45·2	We aim to minimise energy and water consumption through efficient administration, equipment selection, usage and disposal, food storage, preparation and cooking. We have reviewed the methods we use in our preparation, cooking and storage to ensure they are the most efficient and effective means to reduce energy while maintaining quality and freshness. We turn off equipment, heating, lighting and water when not needed and use auto-timers where possible. We use natural ventilation where feasible. We take environmental impact and energy-saving features into consideration when purchasing new equipment (Policy 168).	To reduce water and energy use (Policy 164).
Research and innovation	15	17·9	We work with student groups to support research into studies on behaviour change in dietary choices and implement strategies to promote the uptake of plant-based meals from these studies. We have worked with student and academic research projects to understand how best to encourage behaviour change and the adoption of a reusable cup (Policy 114).	Supporting research and teaching on sustainable food systems and using research to improve university practice (Policy 110).
Procurement	75	89·3	Buying food that is traceable to an identifiable place, and which has been produced using methods that regenerate environmental resources and are respectful of animal and human well-being. Seeking suppliers who can commit to buying an increasing proportion of healthy food from small businesses that use agroecological approaches in urban and rural settings, with the lowest possible ecological impact from transport and storage (Policy 210).	Engage with our supply chain to affect positive economic, environmental and social change (Policy 188).
Nutrition, health and well-being	66	78·6	Fully nutritionally analysed range of grab and go products including sandwiches and salads. • Enabling positive dietary changes by reducing foods high in saturated fat, salt and free sugars and increasing the provision of vegetarian and vegan options plus higher fibre foods, fruit, vegetables and oily fish. Increase the % of food prepared fresh on site. Rapeseed oil to be used for all frying and relevant food production. Vending machines to supply healthy options and to optimise the offer of responsibly sourced hot drinks (Policy 242).	Embed healthy options within menu planning and delivery (Policy 238).
Other Cleaning Growing Deliveries Community Workers	57	67·9	Examples: Eco-friendly cleaners and detergents will be introduced to reduce environmental impact (Policy 104). To utilise green space to grow fresh herbs and thus reduce cost and food miles associated with these products (Policy 192). Work with local and regional suppliers to amalgamate and reduce our deliveries (Policy 130). Becoming involved with offering cookery lessons to local colleges and work experience placements for disadvantaged students as part of our Widening Participation Outreach scheme (Policy 154). Ensure all employees are paid a living wage, and where possible, ensure members of the supply chain are also paid a real living wage (Policy 108).	

Although a thorough qualitative content analysis of the policy commitments was outside the scope of this study, it became evident through the data extraction process that individual commitments for each theme varied considerably in scope, clarity and measurability between universities. Evidence of this variability is illustrated by the detailed and vague FS policy extracts, presented in Table 3. Communication and engagement commitments to encourage sustainable food choices and practices generally included information about the topic being communicated, the intended audience and the mode of communication (Table 4). Topics being communicated were diverse and had relevance to the three pillars of sustainability, including animal welfare, endangered fish species, food safety, healthy eating and nutritional value (social), meat and waste reduction, carbon footprint, local and seasonal produce (environmental) and, to a lesser extent, price (economic). However, few referred to their interconnectedness (e.g. promoting health and planetary benefits of plant-based foods). Many communications were aimed at supporting behaviour change at an institutional or consumer level, promoting sustainable practices to, for example, reduce waste. Effective communication and engagement commitments reached beyond consumers, to engage with the broader food system actors, including external bodies (the Humane Society and Vegan Society), caterers, suppliers and stakeholders. Similarly, they used a variety of communication methods, including directly providing information through newsletters, training, reports, social media and labels and employing indirect approaches (changes to menu hierarchy) to alter consumer behaviour.

**Table 4:** Food sustainability communication commitments

Audience	Topics	Mode
Consumers Community Departments External bodies (e.g. The Humane Society) External caterers Staff and students Stakeholders Suppliers Visitors (virtual and campus)	Acceptability (e.g. prices, menu, quality) Carbon footprint Ethical stance (e.g. animal welfare, endangered species and ecosystems) Food policy Food certification (e.g. Fairtrade, Marine Stewardship Council, Organic), provenance and traceability Food safety (e.g. allergens, ingredients) Healthy eating and nutritional value Links between sustainability and well-being Palm oil Reduction of meat and dairy products Seasonal and local produce Social and environmental sustainability Supply chains Sustainable food practices (e.g. to reduce food waste, conserve water and energy, reduce single-use plastic) and environmental activities Sustainability performance Vegan/plant-based options	Award logo Campaigns and promotions Cooking demonstrations Forums Informational materials at point of sale (e.g. via flyers, notices, labelling, QR codes) Network/working group meetings Newsletters, campus maps and posters Nudges (e.g. menu hierarchy, adoption by default) Reports Social media and website Surveys Training and induction

Food waste commitments were discussed in relation to all but one (making animal feed from former food) of the eight UK food waste strategies^(26)^. Common features of these commitments were to prevent surplus waste (e.g. reduce plate size, use farm-discarded wonky veg, discounts for food close to expiration date), redistribute surplus food (onsite pantry, redistribution apps, student and staff community fridge, community donations) and recycle food via composting or anaerobic digestion for bio-fertilisers and energy recovery, respectively. Less common was institutional commitment to send surplus food waste for processing to make biomaterials (e.g. coffee logs) and to use coffee grounds for land spreading on campus. Institutions also demonstrated commitment to reducing non-food waste (e.g. plastic bottles and disposable food packaging), via targets, actions or achievements throughout the university food system, from suppliers to the end consumer. This included promoting free chilled water fountains, employing pricing strategies (levy on disposable cups and rewards for using reusable cups), providing alternatives to plastic packaging that can be sent for recycling or composting (e.g. aluminium cans and plant-based cutlery, respectively) and reducing transportation packaging on campus and through the supply chain. Together, these commitments provide evidence that many institutions use multidimensional strategies to reduce or eliminate food and non-food waste sent to landfill.

The procurement theme typically consisted of commitments to look beyond economic criteria, referring broadly to social and environmental dimensions of sustainability, or to specific sustainability criteria (traceability, ethical working conditions, sustainable farming practices, human well-being), during the supplier tendering process, when purchasing food or setting franchise agreements. As such, this theme was interlinked with several other themes. First, quality standards and certification commitments, across three domains, food safety, ethics (including environmental and ecosystem protection, animal welfare and workers’ rights) and health (Table 5), were a way of demonstrating institutional commitment to sustainable procurement. Some of the quality standards and certifications applied to the production of goods (e.g. organic), a section of the food sector (farming, e.g. Red Tractor, or marine, e.g. Marine Stewardships Council) or more comprehensively, to the delivery of a healthy, sustainable food service, from farm to fork (e.g. The Sustainable Restaurant Association, Food Made Good accreditation). In contrast, others were applied to specific foods or ingredients (eggs, fish, meat, cocoa, palm oil, soy) and therefore overlapped to some degree with the food- and ingredient-specific themes (fish, palm oil and meat, eggs and dairy products).

**Table 5:** Quality standards and certification

Title	Food safety	Ethical	Health
British Lion eggs	X		
British Retail Consortium	X		
Cocoa life		X	
Compassion in world farming		X	
Eating better		X	
Eat safe award	X		
Environmental stewardship		X	
Fairtrade international		X	
Farm animal welfare committee		X	
Food for the brain accreditation			X
Freedom food /RSPCA – UK		X	
Free Range		X	
Friends of the Earth – Kale yeah! kitchen		X	
Good egg award		X	
ISO standards		X	
Leaf marque		X	
Marine Stewardship Council, Aquaculture Stewardship Council, responsible fishing vessel, FAO: Code of Conduct for Responsible Fisheries		X	
People and Planet League rankings		X	
Quality meat Scotland		X	
Rainforest alliance/union direct trade		X	
Red tractor – UK	X	X	
Round table on responsible soy/palm oil		X	
Soil association:			
Catering mark		X	X
Food for life (green kitchen standard)		X	X
Organic		X	
Sustainable fish cities		X	
Sustainable restaurant association – food made good award		X	X

The food-specific policy commitments went beyond standards and accreditation systems and included commitments to reduce the quantity of high-impact foods (e.g. red meat or overfished species of fish) or production methods (e.g. seine net fishing). Most dominant was the commitment to exclude or reduce ruminant meat and dairy products, often with a pledge to improve the quantity, quality or variety of plant-based alternatives (71·4 %). Comprehensive food-specific policy commitments recognised the need to consider hidden threats to sustainability across the food system (e.g. soy in animal feeds) and went beyond traditional approaches to meat and fish production (e.g. using meat traditionally considered waste or raising awareness of alternative species of fish).

Second, in many cases, procurement commitments focused on building relationships with suppliers, which is likely to be instrumental to a more transformative, just and fair food system. This included relationships with small and medium-sized enterprises and local suppliers, which is consistent with commitments towards increasing the provision of seasonal and local foods, with the potential to generate social and economic benefits for the local community.

The final common FS theme, nutrition, health and well-being, varied considerably but was dominated by content related to the nutritional balance or content of foods and drinks being offered. Policies referred specifically to reducing fat, sugar and salt, exclusion of artificial additives and GM foods, minimising high energy foods and high sugar drinks and increasing the proportion of healthy foods and ingredients, such as sources of healthy fats (oily fish, rapeseed oil). Less common commitments within this theme were pledges to monitor and review the nutritional quality of food provisions, to empower consumers to make healthy food choices (e.g. through provision of nutritional information) and finally, to ensure menus reflected the dietary needs and cultural preferences of the diverse consumers it serves, demonstrating broad commitments to social sustainability.

Despite universities having significant responsibility for research and innovation, less than 20 % of FS policies had a commitment in this area. Research and innovation commitments referred to student and academic projects and research collaborations with internal and external partners. Some policies specified topics of research focus, including health and sustainability, the food system and behaviour change (e.g. encouraging plant-based meals, adoption of reusable cups). Policies that acknowledged how the institutions (student/academic-led) research was being used (e.g. to enhance food service delivery, increase transparency in the university food system and improve monitoring, performance and impact assessment) demonstrated effective integration of research into the food service provisions.

## Discussion

In line with the aims of this research, this comprehensive review of 163 UK universities reports the prevalence of publicly available FS policies and provides insight into the range of FS commitments, including areas of good practice. Approximately half of these universities possessed a publicly available FS policy. This is considerably greater than previously reported for UK institutions^(12,19)^. The sizeable increase in recent years likely reflects the growing urgency and accompanying pressure on institutions to address the climate emergency throughout their activities, namely, research, education and facilities management (including food service provisions). Unfortunately, direct comparison of the policy commitments over time is significantly restricted, as only Hoolohan *et al.* ^(19)^ considered the content of the policies in any detail. Focusing on meat-based commitments, they reported that 54·9 % of FS policies recognised the importance of reducing meat consumption, primarily to tackle the extensive carbon emissions generated from meat production. The UK’s National Food Strategy suggests that aligning national meat consumption with the Scientific Advisory Committee on Nutrition recommendations would achieve ∼43 % of the meat reduction targets required to meet net zero commitments. The National Food Strategy considers this to be a lifestyle change that ‘can hardly be described as a privation’^((27): 109)^, which gravely underestimates the challenges in changing consumer behaviour and neglects the cultural importance of meat and resistance to eating less meat in the UK diet^(28)^.

In this study, meat commitments (often accompanied by pledges to increase plant-based sources of protein) were outnumbered by three of the fifteen FS themes: communication and engagement, food waste and quality standards and certification. This is surprising given the disproportionate contribution of meat to food-related GHG emissions. A similar pattern was observed in a review of New Zealand and Australian universities’ FS policies (*n* 29), with communication and engagement, under the umbrella of community engagement (*n* 8) and food waste prevention (*n* 9), outnumbering animal product (*n* 5) commitments^(29)^. In contrast, they reported a lower prevalence of commitments to quality standards, which may reflect their narrow focus (i.e. Fairtrade accreditation), thereby neglecting the broader standards of ethical procurement. While a greater range of quality standards and certifications were present across the current dataset, few applied to the full food service (production to consumption), therefore unlikely to have a transformative impact on the food systems. The Sustainable Restaurant Association’s ‘Food Made Good Standard’ is a globally recognised sustainable certification that evaluates food services across three pillars (sourcing, society and environment) at every operation level^(30)^ and could provide an opportunity for universities to elevate their commitment to higher social and environmental standards.

Communication and engagement and procurement commitments included soft (information provision) and hard (limiting popular, yet high-impact foods) measures for transitioning towards healthier, more sustainable food services and consumer behaviours in the university setting. Communication, typically favoured over restrictive practices that reduce individual choice, has mostly focused on the provision of information for a single outcome (e.g. improving human health or environmental sustainability), to catering staff and consumers. This is likely a missed opportunity to highlight the combined benefit to human and planetary health of dietary change^(31)^. For example, plant-based diets, rich in fibre and antioxidants, are associated with reduced carbon emissions, natural resource use and risk of non-communicable diseases^(32)^. This is especially important as estimates suggest the UK population consumes less than half of the 30 g recommendation for dietary fibre^(33)^. Furthermore, the limited scope of communication commitments, in most institutions, is unlikely to have a sizeable impact on wider community or stakeholder (including producers) behaviours or practices.

In addition, while a recent systematic review identified that provision of information alone can improve consumer knowledge and attitudes, it has been largely unsuccessful at instigating pro-environmental behaviours^(34)^. In contrast, combined interventions that target both attitudes and contextual factors (barriers and facilitators), including (infra)structures (e.g. opportunities to recycle), education, feedback and enablement (e.g. tools and aids), while making the sustainable option the default, have been effective at changing behaviours. Despite this, much of the policy landscape places emphasis on consumer choice, which Shove^(35)^ argues is for political advantage and is unlikely to be sufficient to promote the societal transformation required for a sustainable food system. In contrast, a more recent ‘Change Points’ approach, ‘developed to enable policy processes to confront the complexities of everyday action, transforming both how problems are framed and how practical initiatives for effecting change are developed’^((36): 1)^, shows promise. This approach is rooted in practice theory and focuses on practices (activities), rather than people’s behaviour. As such, it recognises the connections between production, consumption and wider socio-cultural, political, economic and material developments, which are more conducive to a transformative food systems perspective than individualistic behaviour change approaches.

While research and innovation was the least pervasive FS policy commitment across the current dataset, interventions aimed at supporting healthy, sustainable food choices in the university environment and campus-based sustainable food ‘projects’ are well documented. Across two systematic reviews that evaluated the impact of naturalistic interventions in university outlets, twenty-nine studies reported health outcomes, and twenty-two reported sustainability outcomes^(37,38)^, which aligned with some of the current study themes. Pandy *et al.* ^(38)^ reviewed nudge interventions (*n* 14), primarily focused on changing product characteristics, with outcomes to increase consumption of plant-based foods (*n* 7) and reduce meat consumption (*n* 4), water footprint (*n* 1) and bottled water consumption (*n* 1)^(38)^. Only four (∼29 %) of these studies reported statistically significant improvements with a medium effect size, and the overall effect of nudge interventions was NS. The studies with sustainability outcomes (*n* 8), reviewed by Lee *et al.* ^(37)^, were dominated by behaviour change interventions to reduce waste (50 %), through changes to the environment and the provision of information, with some significant improvements reported.

In contrast to these unremarkable findings, a single case study^(39)^ illustrated how the collective power of interested students and an innovative class project were the catalyst for a garden project that led to sizeable food system change. The authors suggested that the small student-led garden was a stimulus for the institution to grow produce for campus halls, which transformed the local food system. It also documented how students gained valuable graduate-level employability skills, including negotiation skills, securing funding, collaboration with stakeholders, speaking at conferences and hosting educational tours, alongside planning, growing, harvesting and marketing produce at a farmers’ market. Although this approach may not be feasible for many institutions, especially those in urban areas with little space for growing food, other innovative and transformative solutions that channel the collective power of motivated students, inspirational academics and community outreach, alongside effective support from student services and operational staff, could reduce the cognitive-practice gap, first introduced by O’Neill and Sinden^(12)^.

### Strengths and limitations

This is the first study to explore the range of commitments within UK universities’ publicly available FS policies. Despite this, there are limitations that need to be considered when interpreting the findings. First, it is possible that some institutions may house their FS policies internally or include FS commitments within a broader sustainability policy; therefore, omissions may have occurred. Second, the analysis of the textual data extracted from the FS policies was used to provide context and examples, rather than to generate a detailed qualitative analysis, limiting the depth of analysis. Furthermore, the quantitative analysis may give an optimistic picture of universities’ commitments to delivering a sustainable food service as some policy commitments were vague, lacked measurable outcomes or failed to consider the full impact of a specific FS theme. Finally, it was not always possible to determine the focus of some of the FS commitments, meaning that they may not be categorised in a way that reflects the universities’ positionality; for example, provision of free drinking water may be a target for improving hydration (nutrition, health and well-being) or a strategy to reduce the use of plastic bottles (non-food waste).

### Implications for university policy makers and future research

This quantitative content analysis of FS policy commitments has identified areas of good practice and weaknesses that may be of interest to university policy makers and research leaders. Good practice generally reflected commitments that explicitly referred to the interconnectedness of the three pillars of sustainability (social, environment and economic, including potential trade-offs), extended the universities’ reach beyond the campus consumer (including producers, local community, stakeholders and external bodies), were multidimensional (e.g. reducing food waste, while improving food security), spanned the food system (production to consumption) and waste hierarchy (prevention to responsible disposal) and used academic and student-led projects and research to improve the catering service. Weaknesses in the FS policies were related to limitations in scope, clarity and the measurability of commitments. Priorities for future research include (i) holistic evaluation of the impact of measurable FS policy commitments on a combination of health and sustainability outcomes, including any trade-offs, (ii) exploration of barriers and facilitators to transformative policy implementation and (iii) identification of opportunities to further support sector-wide transformation of the food system.

### Conclusion

A growing commitment to sustainable food provisions in UK universities is evident in the rise of publicly available FS polices, compared with previous research. Commitments made in these FS policies reflect the main environmental priorities for reducing the impact of the food system and, to a lesser extent, address key social and economic priorities for delivering an accessible, healthy, sustainable diet. While there were examples of good practice, policy commitments could further benefit from highlighting the alignment between health and sustainable outcomes and improving the clarity, scope and measurability of policy commitments. The latter is needed to allow institutions to evaluate the impact of policy implementation on key outcomes. There were few explicit examples of institutional-research-informed commitments within the FS policies. Furthermore, they appeared to lack transformative capabilities, suggesting there remains a sizeable cognitive-practice gap^(12)^ between institutional research, policy and operational activities. This leaves universities at reputational risk, which may threaten university sustainability, especially given the competitiveness of the current market.

## Supporting information

Blennerhassett et al. supplementary material Blennerhassett et al. supplementary material

## Supplementary material

For supplementary material accompanying this paper, visit https://doi.org/10.1017/S1368980025101055
