# Supplementary material for: University commitments to a sustainable food system: a content analysis of UK higher education institutions food sustainability policies
Source: Public Health Nutr. 2025 Sep 11;28(1):e163. doi: 10.1017/S1368980025101055 (PMC12516621; doi:10.1017/S1368980025101055)
Supplement: Blennerhassett et al. supplementary material [file S1368980025101055sup001.docx]

Supplementary material 1: Appendix A: Content analysis coding template

| **Initial code list** | **Refined code list** | **Evidence** |
| --- | --- | --- |
| **Communication**: commitments to communicate food sustainability messages through the provision of information to a range of different audience | **Communication & Engagement**: communication of food sustainability information was *expanded* to include engagement in dialogue between different actors in the university food system e.g. caterers and suppliers. | Communication of messages such as meat reduction, is crucial to conveying scientific content to support people with sustainable food decisions, as is effective public engagement^(40^**^)^** |
| **Food Waste**: commitment to food waste strategies throughout the university food system, including during storage of foods and ingredients, production of foods on campus, serving food to consumers and disposal of unavoidable food waste. | | Food waste has implications across the three pillars of sustainability. Edible food waste represents financial losses to the consumer and threatens food security. Furthermore, edible food waste, sent to landfill results in avoidable greenhouse gas emissions **^(^**^41)^. |
| **Meat, Dairy & Eggs**: commitments to food sustainability strategies that address the environmental impact of animal source food production, along with ethical and safety (social) concerns related to the current methods of animal-based food production. | | Animal agriculture has a significant impact on greenhouse gas emissions^(16)^, land and water use and biodiversity loss^(18)^ and has implications for animal welfare and human safety^(42)^ |
| **Fish**: commitments to food sustainability strategies that address the environmental impact of fish production, along with ethical and safety concerns related to the current methods of production of fish and fish products | | Marine fishing and overfishing have been implicated in destruction of fish habitat, ecological disruption and by-catch of other marine animals**^(43)^** with implications for community livelihoods**.** In contrast**,** inland fish production pose risk of disease and parasite transfer, residues from chemical and drug use and use of fishmeal to feed farmed fish^(44)^ |
| **Fairtrade**: commitments to purchasing foods, drinks and ingredients that are socially and economically responsible | **Quality Standards and Certification**: commitments to purchasing Fairtrade produce were *extended* to include commitments to other standards of sustainable food production, including those that protect the environment. | Fairtrade has been recognised for its capacity to improve and diversify livelihoods**,** however there are hundreds of food certification and standards that seek to provide consumers with information about the food production process^(45)^ |

Supplementary material 1: Appendix A: Content analysis coding template (cont)

| **Initial code list** | **Refined code list** | **Evidence** |
| --- | --- | --- |
| **Fruit, Vegetables and Plant- Based Ingredients**: commitments to provide a range of affordable fruit, vegetable and other plant-based foods | Full policy coding identified that fruit, vegetables and plant-based commitments covered three distinct areas. This resulted in *refinement* of the template as follows; i. content related to fruit and vegetables as foods and ingredients for health – *moved* to **Nutrition, Health and Well-Being**, ii. **Plant-Based** alternatives to meat and dairy –was *separated* from the original theme, and iii. content related to **local and seasonal** produce was *extended* to include foods and ingredients beyond fruit and vegetables. | Fruit, vegetables and other plant-based foods and ingredients are typically less harmful to the environment^(16)^  Local and seasonal produce is often promoted as sustainable based on reducing GHG emissions associated with food transportation miles and energy (heat and light) savings, when compared to energy needed to produce crops out of season, respectively. Buying locally seasonal produce could also benefit the local economy^(46)^.  Plant-based alternatives to meat and dairy are typically less harmful to the environment^(^**^15)^**. |
|  | **Local and seasonal:** commitments referred to local and seasonal fruit and vegetables, other crops, fish, meat and dairy |  |
|  | **Plant based:**commitment to increasing the affordability, quality and quantity of plant-based alternatives to meat and dairy, *separated* from initial coding for fruit, vegetables and plant-based ingredients for clarity |  |
| **Palm Oil**: commitment to responsible sourcing of palm oil and products containing palm oil through the institutions food system | | Palm oil is implicated in land use conversion of peatlands and tropical forests, resulting in carbon release into the atmosphere and biodiversity loss^(47)^ |
| **Non-Food Waste**: commitment to reduce the amount of packaging generated and wasted through the institutions food system | | While food packaging plays a vital role in food safety and reducing food waste (extending shelf-life and protecting from damage during transportation), virgin fossil-based packaging has a considerable impact on the environment (greenhouse gas emissions, marine microplastics)^(48)^ |

Supplementary material 1: Appendix A: Content analysis coding template (cont)

| **Initial code list** | **Refined code list** | **Evidence** |
| --- | --- | --- |
| **Energy**: commitment to the efficient use of energy resources related to the institutions food system | | Food production and consumption places considerable demand on water and energy resources, through planting and feeding in agriculture and livestock, to cooking at home and within the food and beverage services, including institutions ^(49)^. |
| **Water**: commitment to the efficient use of water resources related to the institutions food system | |  |
| **Research and Innovation**: integration of research and innovative approaches to improve the sustainability of food production and consumption in higher education. | | Research and innovation have been recognised as instrumental in driving the transition to a healthy sustainable food system, through developing and testing solutions, overcoming barriers and uncovering new opportunities^(50)^ |
|  | **Procurement:** commitments to sustainable and responsible food procurement, including social and environmental dimensions of sustainability *added* after the initial coding identified relevant content. | In recognition of the impact of food production and consumption on human and planetary health, the UK government has emphasised that public procurement should consider environmental and social value benefits, alongside economic drivers to keep costs to a minimum, to support a sustainable food production systems^(51)^**.** |
|  | **Nutrition, Health and Well-Being:** commitment to provide a food service that facilitates health and well-being *added* after the initial coding identified relevant content. | Sustainable diets have been defined by the Food and Agriculture Organisation of the United Nations^(52)^ as ‘diets that are nutritionally adequate, healthy, safe, culturally acceptable, economically fair, accessible and affordable, and protective and respectful of biodiversity and ecosystems. |
|  | **Other:** *added* to capture commitments to a sustainable food service that is not addressed by the previous themes |  |
